# Differences in GluN2B-Containing NMDA Receptors Result in Distinct Long-Term Plasticity at Ipsilateral versus Contralateral Cortico-Striatal Synapses

**DOI:** 10.1523/ENEURO.0118-19.2019

**Published:** 2019-11-26

**Authors:** Wei Li, Lucas Pozzo-Miller

**Affiliations:** Department of Neurobiology, University of Alabama at Birmingham, Birmingham, AL 35294

**Keywords:** cortico-striatal synapses, GluN2A, GluN2B, NMDARs, synaptic plasticity

## Abstract

Excitatory neurons in the primary motor cortex project bilaterally to the striatum. However, whether synaptic structure and function in ipsilateral and contralateral cortico-striatal pathways is identical or different remains largely unknown. Here, we describe that excitatory synapses in the mouse contralateral pathway have higher levels of NMDA-type of glutamate receptors (NMDARs) than those in the ipsilateral pathway, although both synapses utilize the same presynaptic vesicular glutamate transporter (VGLUT). We also show that NMDARs containing the GluN2B subunit, but not GluN2A, contribute to this difference. The altered NMDAR subunit composition in these two pathways results in opposite synaptic plasticity induced by θ-burst stimulus: long-term depression in the ipsilateral pathway and long-term potentiation (LTP) in the contralateral pathway. The standard long-term depression (LTD)-inducing protocol using paired postsynaptic and presynaptic activity triggers synaptic depression at ipsilateral pathway synapses, but not at those of the contralateral pathway. Altogether, our results provide novel and unexpected evidence for the lack of bilaterality of NMDAR-mediated synaptic transmission at cortico-striatal pathways due to differences in the expression of GluN2B subunits, which results in differences in bidirectional synaptic plasticity.

## Visual Abstract

**Figure:**
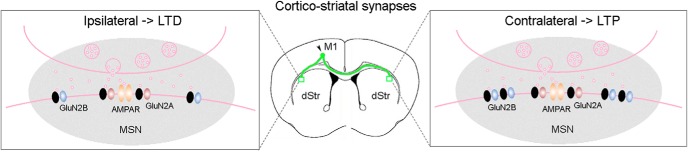

## Significance Statement

Excitatory neurons in the cortex project bilaterally to the striatum. It is generally assumed that synapses formed by ipsilateral and contralateral cortical efferents have similar synaptic features, and serve to synchronize inter-hemispheric activity. We demonstrate that the contralateral cortico-striatal pathway has higher levels of GluN2B-containing NMDARs than the ipsilateral pathway. This altered content of GluN2B and GluN2A subunits in NMDARs results in different forms of long-term potentiation (LTP) and long-term depression (LTD) at excitatory synapses in the ipsilateral and contralateral pathways. These findings provide a novel mechanistic insight into how cortical neurons distinctly modulate ipsilateral and contralateral subcortical regions.

## Introduction

In the excitatory cortico-striatal pathway, glutamatergic synapses express the characteristic complement of AMPA receptors (AMPARs) and NMDA receptors (NMDARs; Shepherd, 2013; Stroebel et al., 2018). NMDARs are heteromultimers comprising two obligatory GluN1 subunits and two modulatory subunits, either GluN2 or GluN3 (Paoletti and Neyton, 2007). There are four GluN2 subunits (A–D) in the brain, but GluN2B and GluN2A predominate in the striatum (Landwehrmeyer et al., 1995; Chapman et al., 2003), where they are present in either heterodimer (GluN1/GluN2B and GluN1/GluN2A) or heterotrimeric combinations (GluN1/GluN2B/GluN2A; Dunah and Standaert, 2003). GluN2B and GluN2A subunits are structurally and functionally distinctive, contributing unique properties to NMDAR function in basal synaptic transmission and plasticity. In addition, these two subunits are expressed at different developmental times: GluN2B is predominant in early postnatal development, whereas the levels of GluN2A progressively increase during development and ultimately exceed those of GluN2B (Monyer et al., 1994). GluN2B-containing NMDARs are preferentially targeted to extrasynaptic sites, while GluN2A-containing NMDARs are localized to the postsynaptic density (Rumbaugh and Vicini, 1999; Gardoni et al., 2006). Notably, GluN2B-containing NMDARs have lower affinity for glutamate, slower channel kinetics, and higher Ca^2+^ permeability (Erreger et al., 2005). GluN2B-containing NMDARs also have a specific binding domain for Ca^2+^/calmodulin-dependent protein kinase II (CaMKII), allowing timely activation of downstream signaling cascades that mediate long-term synaptic strengthening (Strack and Colbran, 1998). All these distinct features imparted to NMDARs by GluN2B and GluN2A subunits account for their different roles in synaptic structure and function (Shipton and Paulsen, 2013). For instance, activation of GluN2B-containing or GluN2A-containing NMDARs in the striatum differentially regulates GABA and glutamate release in target areas (Fantin et al., 2007), and controls glutamate and dopaminergic synaptic transmission (Schotanus and Chergui, 2008). Studies in the hippocampus have suggested that due to the differences in the temporal features of GluN2B-mediated and GluN2A-mediated Ca^2+^ influx, these two subunits have a differential role in the induction of long-term potentiation (LTP) and long-term depression (LTD; Shipton and Paulsen, 2013).

Morphologic and behavioral studies have long demonstrated that cortical neurons send bilateral projections to multiple subcortical regions (Fame et al., 2011). It is generally assumed that synapses formed by ipsilateral and contralateral cortical efferents have similar postsynaptic features, and serve to synchronize inter-hemispheric activity. However, this assumption has not been directly examined. Expression of light-sensitive cation channels in cortical projection neurons allows their selective stimulation in only one hemisphere and the characterization of potential differences between ipsilateral and contralateral cortico-striatal synaptic transmission and plasticity. We uncovered the lack of bilaterality of NMDAR-mediated synaptic transmission at cortico-striatal pathways due to differences in the expression of GluN2B subunits, which results in differences in bidirectional synaptic plasticity.

## Materials and Methods

### Animals

*Drd1a*-tdTomato [B6.Cg-Tg(Drd1a-tdTomato)6Calak/J] (https://www.jax.org/strain/016204) mice were purchased from The Jackson Laboratory; *Drd2*-EGFP [Tg(Drd2-EGFP)S118Gsat] (http://www.informatics.jax.org/allele/MGI:3843608) mice were purchased from the Mutant Mouse Resource and Research Centers. All mice were maintained on a C57BL/6J background, and kept on a 12/12 h light/dark cycle with food and water *ad libitum*. Male mice (P40–P70) were used for all the experiments. All animal procedures were performed in accordance and after approval by the institutional animal care and use committee.

### Stereotaxic injections

All adeno-associated viruses (AAVs) were obtained from the UNC Vector Core, and delivered via stereotaxic intracranial injections. Male mice (P20–P27) were anesthetized with 4% isoflurane in 100% oxygen gas; anesthesia was maintained with 1–2.5% isoflurane. Mice were placed in a stereotactic frame (David Kopf Instruments), and their body temperature was maintained with a heating pad. The scalp was shaved and then sterilized with 70% ethanol. A rostral-caudal incision was made to access the skull, a hole was drilled, and virus was delivered through a 2.5-µl syringe (Hamilton Company) at a rate of 0.25 µl/min using a microsyringe pump (UMP3 UltraMicroPump, Micro4, World Precision Instruments). For optogenetic stimulation of cortical axons, AAV-CaMKIIα-ChR2 (H134R)-EYFP was unilaterally injected into the primary motor cortex (M1; AP = +1.0 mm, ML = +1.5 mm, DV = –1.2 mm; 0.5–1 µl per site). For dual color optogenetic activation of ipsilateral and contralateral cortical inputs to the same striatal slice, AAV-Syn-Chrimson-tdTomato was injected into the ipsilateral M1, and AAV-Syn-Chronos-GFP into the contralateral M1. For optogenetic stimulation of cortical axons with high-frequency θ-burst patterns, AAV-CaMKIIα-ChR2 (E123T/T159C)-EYFP (ChETA) was injected into M1. Following the injections, the incision was closed with surgical glue. Topical antibiotic ointment (bacitracin zinc, neomycin sulfate, and polymyxin B sulfates; Actavis) was applied to the incision, and carprofen (5 mg/kg; Zoetis) was administered intraperitoneally. Mice were used for experiments after three to four weeks for the viral expression of CaMKIIα-ChR2 (H134R), Syn-Chrimson, and Syn-Chronos, and five to six weeks for the expression of CaMKIIα-ChETA. The spread of opsin expression was 30.7 ± 10.3% of the entire M1 area (*n* = 10).

### *Ex vivo* brain slices

Male mice were deeply anesthetized with a mixture of ketamine (100 mg/kg) and xylazine (10 mg/kg), and transcardially perfused with ice-cold low Na^+^, low Ca^2+^ “cutting” artificial CSF (aCSF) containing the following: 87 mM NaCl, 2.5 mM KCl, 0.5 mM CaCl_2_, 7 mM MgCl_2_, 1.25 mM NaH_2_PO_4_, 25 mM NaHCO_3_, 25 mM glucose, and 75 mM sucrose, bubbled with 95% O_2_/5% CO_2_. The brain was rapidly removed, and coronal (for optogenetic stimulation) or parasagittal (for electrical stimulation) slices were cut at a thickness of 300 µm with a vibrating blade microtome (VT1200S, Leica Biosystems). Coronal slices were surgically cut in the midline to separate slices ipsilateral to the AAV injection from those contralateral to the AAV injection. Slices were then transferred to normal aCSF containing the following: 130 mM NaCl, 3.5 mM KCl, 2 mM CaCl_2_, 2 mM MgCl_2_, 1.25 mM NaH_2_PO_4_, 24 mM NaHCO_3_, and 10 mM glucose, bubbled with 95% O_2_/5% CO_2_, at 32°C for 30 min and allowed to recover for 1 h at room temperature before recordings.

### Intracellular recordings

Individual slices were transferred to a submerged chamber mounted on a fixed-stage upright microscope (Zeiss Axioskop FS) and continuously perfused at room temperature with normal aCSF containing the following: 0 µM MgCl_2_, 50 µM picrotoxin (Millipore Sigma, catalog #P1675), and 10 µM glycine (Millipore Sigma, catalog #8898). Slices were visualized by infrared differential interference contrast microscopy with a water-immersion 63× objective (0.9 NA, Zeiss). D1 and D2 medium spiny neurons (MSNs) were identified by fluorescence excitation of tdTomato and EGFP, respectively (554 nm and 470 nm; X-Cite Turbo, Excelitas Technologies). Recordings were acquired with Axopatch-200B amplifiers (Molecular Devices), filtered at 2 kHz, and digitized at 10 kHz with an ITC-18 A/D-D/A interface (Instrutech) controlled by custom-written software in a G5 PowerMac Apple computer (TI-WorkBench, provided by Dr. Takafumi Inoue; Inoue, 2018). Input resistance was measured with hyperpolarizing voltage pulses (50 ms, 20 mV). Cells with series resistances above 15 MΩ were discarded, and cells were also excluded if any whole-cell parameter (i.e., Cm, Ri, Rs) changed by ≥20% during the recordings.

Whole-cell voltage-clamp recordings were performed with unpolished pipettes (World Precision Instruments), containing the following: 120 mM Cs-gluconate, 17.5 mM CsCl, 10 mM Na-HEPES, 4 mM Mg-ATP, 0.4 mM Na-GTP, 10 mM Na_2_-creatine phosphate, 0.2 mM Na-EGTA, and 5 mM QX-314 (290–300 mOsm, pH 7.3, final resistance: 3–4 MΩ). Total EPSCs were recorded at –50 mV in MSNs, and NMDAR EPSCs were then isolated by addition of the AMPAR antagonist 2,3-dioxo-6-nitro-1,2,3,4-tetrahydrobenzo[*f*]quinoxaline-7-sulfonamide (NBQX, 10 µM; Tocris, catalog #1044). In some experiments, MSNs were recorded at +60 mV in the presence of normal Mg^2+^. NMDAR EPSCs were confirmed by addition of the NMDAR antagonist D,L-APV (20 µM; Tocris, catalog #0106) at the end of the recordings. To evoke EPSCs in MSNs by electrical stimulation, a θ glass electrode (World Precision Instruments) was placed on the cortical layer VI close to the corpus callosum, and 100 µs-long stimuli were delivered every 20 s. To evoke EPSCs by optogenetic stimulation of ChR2 or ChETA, blue light from a fiber optic-coupled LED (470 nm, Thorlabs; 0.5- to 1.5-ms duration, 5–8 mW/mm^2^) was delivered to either ipsilateral or contralateral MSNs by a LED driver (LEDD1B, Thorlabs). For dual color optogenetic stimulation of Chrimson and Chronos in the same slice, red light from a fiber optic-coupled LED (625 nm, Thorlabs; 1–4 mW/mm^2^) was used for excitation of ipsilateral Chrimson, and blue light from a laser-LED hybrid source (470 nm, X-Cite Turbo) was used for excitation of contralateral Chronos through the 63× microscope objective. Based on the spectral properties of Chronos and Chrimson (Klapoetke et al., 2014), and to minimize potential “bleed-through” between the two opsins during 2-color light stimulation, we systematically chose the minimum intensity and the shorter pulse duration of blue light that were needed to evoke EPSCs by activation of Chronos-expressing presynaptic axons (see details in Phillips et al., 2019). The selective GluN2B antagonist ifenprodil (3 µM; Millipore Sigma, catalog #I2892) was perfused for 10 min, followed by addition of the selective GluN2A antagonist TCN-201 (2 µM; Millipore Sigma, catalog #SML0416) for an additional 10 min. We chose 10 min for sequential pharmacological treatments because the effects of ifenprodil and TCN-201 have reached equilibrium at that time.

For induction of cortico-striatal synaptic plasticity by optogenetic stimulation of cortical axons with θ-burst stimulation (TBS) patterns or spiking-timing dependent plasticity (STDP) pairing, whole-cell current-clamp recordings of EPSPs were performed with pipettes containing the following: 140 mM K-gluconate, 5 mM KCl, 10 mM Na-HEPES, 4 mM Mg-ATP, 2 mM MgCl_2_, 0.3 mM Na-GTP, 10 mM Na_2_-creatine phosphate, and 0.2 mM Na-EGTA (290–300 mOsm, pH 7.3, final resistance: 3–4 MΩ). Baseline EPSP amplitudes were obtained with single blue light stimuli (470 nm, Thorlabs; 0.5- to 1.5-ms duration, 5–8 mW/mm^2^) delivered every 30 s to either ipsilateral or contralateral MSNs at their resting membrane potential (about –80 mV). MSNs were depolarized to –70 mV during the induction phase of synaptic plasticity. TBS pattern of blue light pulses consisted of 10 trains of 10 bursts, each burst having 4 pulses at 100 Hz, with 200 ms between bursts, and 10 s between trains (Park et al., 2014). The STDP pairing protocol consisted of 20 trains of 5 bursts at 1 Hz, each burst having three pulses at 5 Hz; each burst included three spikes induced by current injections into MSNs (2 ms, 2 nA), which was followed by a single light stimulus with 10 ms of delay (Wu et al., 2015). Pretreatment with ifenprodil or TCN-201 was used to test the requirement of GluN2B and GluN2A in ipsilateral and contralateral cortico-striatal synaptic plasticity. The CB1 receptor antagonist AM-251 (5 µM; Tocris, catalog #1117) was used to test the requirement of CB1 receptors in LTD in the ipsilateral pathway.

### Immunofluorescence

Male mice were anesthetized with a ketamine and xylazine mixture, and transcardially perfused with 4% paraformaldehyde in PBS. Brain samples were then postfixed in 4% paraformaldehyde overnight at 4°C. Sections were cut at 60 µm using a vibrotome, permeabilized with 0.25% Triton X-100 for 2 h, and blocked with 10% normal serum for 1 h. Sections were incubated overnight at 4°C with anti-rabbit tdTomato (1:2000; TaKaRa, catalog #632543, RRID:AB_2307319), anti-rabbit GFP (1:2000; Abcam, catalog #ab290, RRID:AB_2313768), anti-chicken GFP (1;2000; Abcam, catalog #ab13970, RRID:AB_300798), anti-rabbit mCherry (1:2000; Abcam, ab167453, RRID:AB_2571870), anti-guinea pig vesicular glutamate transporter 1 (VGLUT1) (1:500; Millipore Sigma, catalog #AB5905, RRID:AB_2301751), anti-rabbit VGLUT2 (1:500; Millipore Sigma, catalog #V-2514, RRID:AB_477611) primary antibodies. After the primary antibodies, sections were rinsed with PBS three times each 10 min and incubated for 2 h at room temperature with corresponding secondary antibodies tagged with Alexa Fluor 488, 594, or 647 (Jackson ImmunoResearch). Sections were coverslipped with Vectashield mounting media (Vector Laboratories). All images were acquired using 10× (0.3 NA), 20× (0.8 NA), or 63× (1.4 NA) objectives in an LSM-800 Airyscan confocal microscope (Zeiss).

### Statistical analyses

All data were analyzed using Prism (GraphPad). Differences between groups were tested using either two-tailed unpaired Student’s *t* test (two groups) or one-way ANOVA (more than two groups). Repeated measures two-way ANOVA with the Bonferroni *post hoc* test was used for comparing the effects of pharmacological treatments and stimulation of either ipsilateral or contralateral cortico-striatal pathways. All data are shown as mean ± SE, with *n* and specific statistical test as described in the figures or text. Differences were considered statistically significant at *p < 0.05, **p < 0.01, and ***p < 0.0001. Statistical power was calculated with G*Power (Faul et al., 2009).

## Results

**Figure 1.**
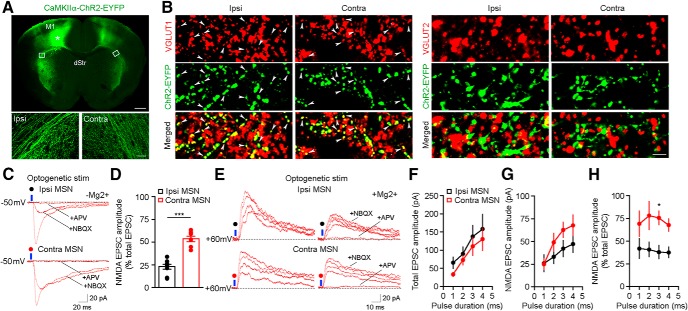
Morphologic and functional features of ipsilateral and contralateral cortico-striatal pathways. ***A***, EYFP immunostaining shows the bilateral projection of M1 cortical neurons in the striatum. Asterisk shows the injection site of AAV-CaMKIIα-ChR2-EYFP in M1; white boxes in the ipsilateral and contralateral dorsolateral striatum are enlarged below. Scale bars: 500 µm (top) and 50 µm (bottom). ***B***, Double VGLUT1 and EYFP immunostaining (left) shows co-localized puncta (arrowheads) in the striatum of both hemispheres, ipsilateral and contralateral to the AAV-CaMKIIα-ChR2-EYFP injection site in M1. Double VGLUT2 and EYFP immunostaining (right) shows a lack of co-localization. Scale bar: 5 µm. ***C***, Representative EPSCs (from ***D***) evoked by blue light (470 nm) in ipsilateral (top) and contralateral (bottom) MSNs in striatal slices from mice expressing CaMKIIα-ChR2-EYFP in M1. Traces are from baseline, 10 min after NBQX, and 10 min after D,L-APV. ***D***, Average of the NMDAR component of the blue light-evoked EPSC in ipsilateral and contralateral MSNs (*n* = 9, Ipsi MSNs; *n* = 7, Contra MSNs; *p* < 0.0001, unpaired Student’s *t* test). ***E***, Representative EPSCs (from ***F–H***) evoked by blue light with different durations (1, 2, 3, and 4 ms) in ipsilateral (top) and contralateral (bottom) MSNs. MSNs were held at +60 mV and perfused with aCSF containing normal Mg^2+^. Traces are from baseline, 10 min after NBQX, and 10 min after D,L-APV. ***F–H***, I/O relationship between light pulse duration, and total EPSC amplitude (***F***; *n* = 6, Ipsi MSNs; *n* = 6, Contra MSNs; *p* = 0.14, two-way ANOVA with Bonferroni *post hoc* tests) or NMDAR EPSC amplitude (***G***; *p* = 0.036) or the ratio of NMDAR EPSC amplitude to total EPSC amplitude (***H***; *p* < 0.0001).

To selectively recruit ipsilateral or contralateral cortical efferents onto striatal MSNs, we injected AAVs into one hemisphere of the M1 for the expression of CaMKIIα-driven channelrhodopsin-2 (ChR2) in excitatory cortical neurons (Fig. 1*A*, asterisk). Studies have identified two types of cortico-striatal projection neurons: intratelencephalic (IT) neurons that project bilaterally to the striatum, and pyramidal tract (PT) neurons that project ipsilaterally to it (Shepherd, 2013). Our results present a mixed response in MSNs induced by afferents derived from these two groups of cortical neurons. Immunostaining for the marker EYFP demonstrates that, in addition to a dense innervation in the ipsilateral striatum, labeled M1 neurons also send a decussating callosal projection to the contralateral striatum. In the striatum, MSNs receive diverse excitatory inputs that express either vesicular glutamate transporter (VGLUT)1 or VGLUT2 in their presynaptic terminals (Fremeau et al., 2001; Herzog et al., 2001). Double immunostaining for either VGLUT1 or VGLUT2 and EYFP showed that VGLUT1, but not VGLUT2, co-localized with EYFP in the ipsilateral and contralateral pathways (Fig. 1*B*), indicating that both cortical pathways use the same presynaptic vesicular glutamate transporter.

To pharmacologically isolate EPSCs mediated by NMDARs in MSNs, blue light (470 nm) was delivered to coronal striatal slices perfused with Mg^2+^-free aCSF containing the NMDAR modulator glycine, the AMPAR antagonist NBQX, and the GABA_A_R antagonist picrotoxin (Fig. 1*C*). NMDAR-mediated EPSCs were confirmed by their complete blockade by the NMDAR antagonist D,L-APV. We compared the ratio of the amplitude of NMDAR-mediated EPSCs to that of total EPSCs in ipsilateral MSNs versus contralateral MSNs. Unexpectedly, the fraction of the ESPC amplitude mediated by NMDARs (normalized to the total EPSC amplitude) is significantly larger in contralateral (53.5 ± 3.2%) than in ipsilateral (22.9 ± 2.7%) MSNs (Fig. 1*D*). Analysis of EPSC kinetics showed that the decay time was longer in contralateral (95.78 ± 9.6 ms) than in ipsilateral (69.4 ± 6.2 ms) MSNs. To characterize the NMDAR-mediated EPSCs in the presence of normal Mg^2+^, MSNs from the ipsilateral or contralateral pathways were held at +60 mV, and EPSCs evoked by blue light (1–4 ms) were recorded before and after NBQX application (Fig. 1*E*). Input/output (I/O) curves showed that the amplitude of total EPSCs in response to different light pulse durations was not different between two pathways (Fig. 1*F*), but the amplitude of NMDAR-mediated EPSCs and the ratio of that to the amplitude of total EPSCs were significantly higher in the contralateral pathway (Fig. 1*G*,*H*).

**Figure 2.**
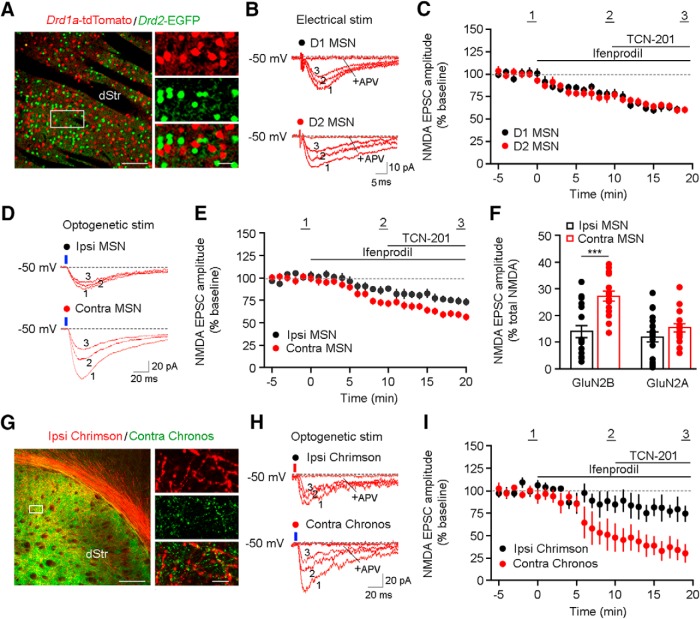
Differences in subunit composition of synaptic NMDARs between the ipsilateral and contralateral cortico-striatal pathways. ***A***, Double tdTomato and EGFP immunostaining shows segregation of D1 and D2 MSNs in the striatum of *Drd1a*-tdTomato::*Drd2*-EGFP double transgenic mice. ***B***, Representative NMDAR EPSCs (from ***C***) in D1 (top) and D2 MSNs (bottom) evoked by electrical stimulation of the cortico-striatal pathway. Traces are from baseline (1), 10 min after ifenprodil (2), 10 min after TCN-201 (3), and 10 min after D,L-APV. ***C***, Average effects of ifenprodil, TCN-201, and D,L-APV on NMDAR EPSC amplitudes (*n* = 7, D1 MSNs; *n* = 6, D2 MSNs; *p* = 0.56, two-way ANOVA). ***D***, Representative EPSCs (from ***E***) evoked by blue light in ipsilateral (top) and contralateral (bottom) MSNs. Traces are from baseline (1), 10 min after ifenprodil (2), and 10 min after TCN-201 (3). ***E***, Average effects of ifenprodil and TCN-201 on blue light-evoked NMDAR EPSCs (*n* = 19, Ipsi MSNs; *n* = 16, Contra MSNs; *p* = 0.0013, two-way ANOVA). ***F***, Average percentage of the ifenprodil-sensitive and TCN-201-sensitive components of NMDAR-mediated EPSCs. The ifenprodil-sensitive component of the NMDAR EPSCs was calculated by subtracting EPSCs after 10 min in ifenprodil from baseline EPSCs; the TCN-201-sensitive component of the NMDAR EPSCs was calculated by subtracting EPSCs after 10 min in TCN-201 from EPSCs in the presence of ifenprodil (***p < 0.0001, unpaired Student’s *t* test). ***G***, tdTomato and GFP immunostaining shows ipsilateral Chrimson expression and contralateral Chronos expression. Scale bars: 100 µm (left) and 10 µm (right). ***H***, Representative EPSCs (from ***I***) evoked by either Chrimson-activating red light (625 nm, top) or Chronos-activating blue light (470 nm, bottom). Traces are from baseline (1), 10 min after ifenprodil (2), and 10 min after TCN-201 (3). ***l***, Average effects of ifenprodil and TCN-201 on red light-evoked and blue light-evoked NMDAR EPSCs (*n* = 19, Ipsi MSNs; *n* = 16, Contra MSNs; *p* = 0.028, two-way ANOVA).

Because GluN2B and GluN2A subunits are the major components of NMDARs in the striatum (Landwehrmeyer et al., 1995; Chapman et al., 2003), and the striatum contains two distinct types of neurons, D1-expressing MSNs and D2-expressing MSNs (Gerfen and Surmeier, 2011), we examined the properties conferred to NMDARs by these two subunits at cortico-striatal synapses in *ex vivo* slices from double transgenic mice expressing tdTomato in D1 neurons and EGFP in D2 neurons (*Drd1a*-tdTomato::*Drd2*-EGFP; Fig. 2*A*). We first recorded pharmacologically isolated NMDAR-mediated EPSCs in MSNs evoked by electrical stimulation of M1 layer VI close to the corpus callosum. The amplitude of NMDAR-mediated EPSCs was reduced by the selective GluN2B antagonist ifenprodil, and further reduced by addition of the GluN2A antagonist TCN-201 (Fig. 2*B*). The fractional reductions by ifenprodil and TCN-201 were comparable in D1 MSNs and D2 MSNs (Fig. 2*C*). To more unequivocally demonstrate if the presence of GluN2B and/or GluN2A subunits is responsible for the difference in the amplitude of NMDAR-mediated EPSCs in the ipsilateral and contralateral pathways, we used *ex vivo* brain slices from mice expressing ChR2 in M1 of one brain hemisphere. The reductions of NMDAR-mediated EPSCs by sequential application of ifenprodil and TCN-201 were significantly larger in MSNs contralateral to the ChR2-expressing M1 than in those MSNs ipsilateral to the labeled M1 (Fig. 2*D*,*E*). In addition, the fraction of the EPSC mediated by GluN2B-contaning NMDARs was significantly larger in contralateral MSNs (*p* < 0.0001), while that of the EPSC mediated by GluN2A-contaning NMDARs was not (*p* = 0.18; Fig. 2*F*). To directly compare ipsilateral and contralateral cortical inputs on MSNs of the same striatal slice, we expressed the blue light-activated opsin Chronos in M1 of one hemisphere and the red light-activated opsin Chrimson in the opposite M1, and recorded NMDAR EPSCs in MSNs in one side of the striatal slice (Fig. 2*G*). Consistently, the effects of sequential application of ifenprodil and TCN-201 on the amplitude of NMDAR-mediated EPSCs were larger for blue light-stimulated contralateral efferents than for red light-stimulated ipsilateral efferents, and that reduction is due to NMDARs containing GluN2B subunits (Fig. 2*H*,*l*).

**Figure 3.**
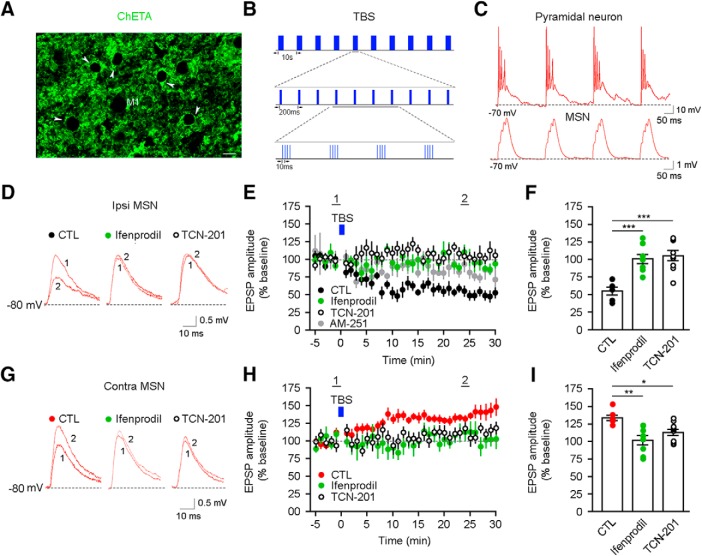
The role of GluN2A-containing and GluN2B-containing NMDARs in TBS-induced synaptic plasticity at the ipsilateral and contralateral cortico-striatal pathways. ***A***, EYFP immunostaining shows ChETA expression in M1. Arrowheads indicate soma of pyramidal neurons expressing ChETA. Scale bars: 10 µm. ***B***, Schematic diagram of TBS. ***C***, TBS pattern of blue light (470 nm) pulses evokes spiking in a ChETA-expressing M1 pyramidal neuron (top) and temporally summating subthreshold EPSPs in a MSN (bottom). ***D***, Representative EPSPs (from ***E***) evoked by a single blue light pulse before (1) and 25 min after TBS of blue light pulses (2) in control or slices treated with either ifenprodil or TCN-201. ***E***, TBS of blue light (bar) induced LTD of EPSPs in ipsilateral MSNs, which was blocked by either ifenprodil or TCN-201 (*n* = 7, CTL; *n* = 8, ifenprodil; *n* = 9, TCN-201; *p* < 0.0001 CTL vs ifenprodil and TCN-201, two-way ANOVA). LTD in ipsilateral MSNs was sensitive to AM-251 treatment (*n* = 5; *p* < 0.0001 CTL vs AM-251, two-way ANOVA). ***F***, Analysis of EPSP amplitude at 25–30 min after TBS in ipsilateral MSNs (*p* < 0.0001 CTL vs ifenprodil and TCN-201, one-way ANOVA). ***G***, Representative EPSPs (from ***H***) evoked by a single blue light pulse before (1) and 25 min after TBS of blue light pulses (2) in control or slices treated with either ifenprodil or TCN-201. ***H***, TBS of blue light (bar) induced LTP of EPSPs in contralateral MSNs, which was blocked by either ifenprodil or TCN-201 (*n* = 7, CTL; *n* = 7, ifenprodil; *n* = 10, TCN-201; *p* = 0.0023 ifenprodil vs CTL; *p* = 0.0049 TCN-201 vs CTL; two-way ANOVA). ***I***, Analysis of EPSP amplitude at 25–30 min after TBS in contralateral MSNs (*p* < 0.01 CTL vs ifenprodil; *p* < 0.05 CTL vs TCN-201, one-way ANOVA). *p < 0.05, **p < 0.01, ***p < 0.0001.

**Figure 4.**
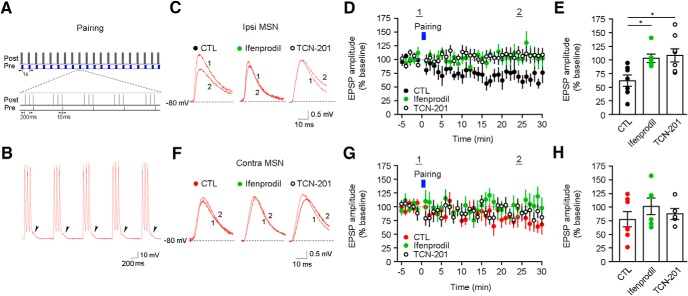
The role of GluN2A-containing and GluN2B-containing NMDARs in postsynaptic-presynaptic STDP pairing-induced synaptic plasticity at the ipsilateral and contralateral cortico-striatal pathways. ***A***, Schematic diagram of STDP protocol, where postsynaptic depolarization is followed by presynaptic light stimulus. ***B***, Representative trace shows spikes induced by postsynaptic current injections and subsequent EPSPs (arrowheads) induced by delayed presynaptic stimulus. ***C***, Representative EPSPs (from ***D***) evoked by a single blue light pulse before (1) and 25 min after paring (2) in control or slices treated with either ifenprodil or TCN-201. ***D***, STDP pairing (bar) induced LTD of EPSPs in ipsilateral MSNs, which was blocked by either ifenprodil or TCN-201 (*n* = 7, CTL; *n* = 6, ifenprodil; *n* = 8, TCN-201; *p* = 0.034 CTL vs ifenprodil; *p* = 0.029 CTL vs TCN-201; two-way ANOVA). ***E***, Analysis of EPSP amplitude at 25–30 min after pairing in ipsilateral MSNs (*p* < 0.05 CTL vs ifenprodil; *p* < 0.05 CTL vs TCN-201; one-way ANOVA). ***F***, Representative EPSPs (from ***G***) evoked by a single blue light pulse before (1) and 25 min after pairing (2) in control or slices treated with either ifenprodil or TCN-201. ***G***, Pairing (bar) did not induce synaptic plasticity in contralateral MSNs, and ifenprodil or TCN-201 had no effect on EPSPs (*n* = 7, CTL; *n* = 6, ifenprodil; *n* = 5, TCN-201; *p* = 0.29 CTL vs ifenprodil; *p* = 0.66 CTL vs TCN-201; two-way ANOVA). ***H***, Analysis of EPSP amplitude at 25–30 min after pairing in contralateral MSNs (*p* > 0.05 CTL vs ifenprodil; *p* > 0.05 CTL vs TCN-201; one-way ANOVA). *p < 0.05.

We next tested whether different levels of GluN2B-contaning and GluN2A-contaning NMDARs at ipsilateral and contralateral cortico-striatal synapses could result in distinct synaptic plasticity properties. To selectively activate axons from M1 pyramidal neurons from only one hemisphere with TBS light patterns, we use ChETA because it follows high-frequency light pulse trains with less inactivation than ChR2 (Xie et al., 2013). ChETA was expressed in cortical pyramidal neurons in M1 (Fig. 3*A*). TBS pattern of blue light pulses reliably activated presynaptic ChETA-expressing fibers and resulted in temporally summating subthreshold EPSPs in MSNs (Fig. 3*B,C*). Surprisingly, blue-light TBS of cortical inputs to ipsilateral MSNs resulted in LTD of EPSP amplitudes, which was blocked by either ifenprodil or TCN-201 (Fig. 3*D–F*). The TBS-LTD is dependent on CB1 receptors, as the antagonism of these receptors by AM-251-blocked LTD (Fig. 3*E*). However, blue-light TBS of cortical inputs to contralateral MSNs resulted in LTP of EPSP amplitudes, which was also blocked by either ifenprodil or TCN-201 (Fig. 3*G–I*). We next examined LTD in ipsilateral and contralateral MSNs by using a STDP protocol, where postsynaptic current injection into MSNs precedes presynaptic blue light stimulation of ChETA-expressing cortical afferents (Fig. 4*A*,*B*). Such “postsynaptic-presynaptic” STDP protocol resulted in a LTD of EPSP amplitudes in ipsilateral MSNs that is sensitive to ifenprodil and TCN-201 (Fig. 4*C–E*), similar to TBS-induced LTD. However, neither LTD nor LTP was induced by this protocol in contralateral MSNs (Fig. 4*F*–*H*). Blockade of GluN2B and GluN2A subunits did not alter EPSP amplitudes in the contralateral pathway. These data demonstrate that GluN2B-contaning and GluN2A-contaning NMDARs are both required for various forms of LTP and LTD in ipsilateral and contralateral cortico-striatal pathways. However, the different content of synaptic NMDAR subunits in each pathway and the extent of recruitment of these subunits during the induction phase of synaptic plasticity seem to influence the direction of long-term synaptic plasticity.

## Discussion

In this study, we provide the first evidence that the glutamatergic synapses in the contralateral cortico-striatal pathway contain higher levels of GluN2B-containing NMDARs than those in the ipsilateral pathway. We also demonstrate that such distinct subunit composition is associated with TBS-LTP at contralateral cortico-striatal synapses, but TBS-LTD at ipsilateral cortico-striatal synapses using the same pattern of optogenetic stimulation of ChETA-expressing axons of M1 pyramidal neurons. A postsynaptic-presynaptic STDP protocol induces LTD at ipsilateral synapses, but has no effect on contralateral synapses.

The amplitude of intracellular Ca^2+^ levels triggered by influx through NMDARs dictates the induction of LTP and LTD, with higher Ca^2+^ levels promoting LTP (Shouval et al., 2002). Consistently, we observed that the levels of NMDARs in the cortico-striatal pathways correlate with the direction of synaptic plasticity, with TBS-LTP in the contralateral pathway that express higher NMDAR levels, and TBS-LTD and STDP-LTD in the ipsilateral pathway that express lower NMDAR levels. In addition, we found that such distinct consequences are mainly due to different expression levels of GluN2B but not GluN2A. Studies in the hippocampus have suggested that the differential contribution of GluN2A-containing or GluN2B-containing NMDARs to LTP and LTD varies, depending on the stimulus paradigm, developmental stage, and the specificity and concentration of the pharmacological antagonists used (Shipton and Paulsen, 2013). An early study in the hippocampus showed that antagonism of GluN2A-containing NMDARs prevented the induction of high-frequency stimulation-induced and pairing-induced LTP, but antagonism of GluN2B-containing NMDARs prevented the induction of LTD by low-frequency afferent stimulation (Liu et al., 2004). In young rats, both LTP and LTD were reduced by antagonism of GluN2A-containing NMDARs, but only LTP was decreased by antagonism of GluN2B-containing NMDARs (Bartlett et al., 2007). Moreover, STDP-LTP, but not high-frequency stimulation-induced LTP, was prevented by an antagonist of GluN2B-containing NMDARs (Zhang et al., 2008); however, higher concentration of this antagonist also impaired TBS-induced LTP (Volianskis et al., 2013). Genetic studies show that enhancing GluN2B expression in the hippocampus led to an increase in LTP and not LTD (Tang et al., 1999; Wang et al., 2009), while GluN2B knockout or RNAi-mediated GluN2B knockdown resulted in an impairment of LTP and LTD (Kutsuwada et al., 1996; Akashi et al., 2009; Foster et al., 2010). In addition, genetic deletion of GluN2A subunits resulted in a reduction in LTP and LTD amplitude in the dentate gyrus (Kannangara et al., 2015). In the striatum, GluN2B and GluN2A subunits are thought to differentially shape the time window for the induction of LTP and LTD during STDP (Evans et al., 2012). Our results demonstrate that higher levels of GluN2B-containing NMDAR at contralateral cortico-MSNs synapses are associated with TBS-LTP, whereas lower levels of these receptors at ipsilateral cortico-striatal synapses are associated with TBS-LTD and STDP-LTD. Our findings also suggest although the levels of GluN2B-containing NMDAR may define the polarity of plasticity, NMDAR containing both subunits are still needed for LTP and LTD, as antagonists of GluN2B-containing or GluN2A-containing NMDARs prevent long-term changes in EPSP amplitude after afferent stimulation. In addition to GluN1/GluN2A and GluN1/GluN2B, there exists GluN1/GluN2A/GluN2B in the striatum (Li et al., 2004). The role of NMDAR triheteromers needs to be further explored, because the pharmacological properties of the triheteromers substantially distinguish from those of the diheteromers (Stroebel et al., 2018).

Induction of long-term synaptic plasticity in striatal slices has been challenging (Lovinger, 2010). The high-frequency stimulation pattern used to induce LTP often results in the induction of LTD, and vice versa. This difficulty has been ascribed to an excessive blockade of NMDARs by extracellular Mg^2+^, and the alteration of intracellular signaling during whole-cell recordings, as experiments in extracellular solution with low Mg^2+^ and the use of extracellular field recordings improve the success rate of LTP and LTD induction. Our results provide an additional explanation for the inconsistent observations of plasticity in striatal MSNs. Electrical stimulation inevitably results in the stimulation of axons coming from the ipsilateral and the contralateral primary motor cortex, which confounds the results due to the different levels of GluN2B-containing NMDARs in each pathway. In this condition, the direction of plasticity is uncertain, in contrast to clear optogenetic-induced plasticity.

NMDAR subunits in the striatum are modulated by dopaminergic inputs from the substantia nigra pars compacta (SNc; Surmeier et al., 2007; Gerfen and Surmeier, 2011). The activation of dopamine receptors in the striatum mediates distinct membrane trafficking of GluN2B and GluN2A (Hallett et al., 2006). These two subunits also play a different role in dopamine receptor-mediated alterations of dendritic spine morphology in MSNs; Vastagh et al., 2012). Genetic deletion or pharmacological inhibition of GluN2A in the striatum facilitates dopamine receptor-mediated potentiation of NMDA responses, whereas inhibition of GluN2B prevents such potentiation (Jocoy et al., 2011). Studies in the hippocampus further suggest a direct interaction of D1 receptors with GluN2A-containing NMDARs, and D2 receptors with GluN2B-containing NMDARs in a specific complex (Lee et al., 2002; Liu et al., 2006). Evidence also suggests that activation of D1 receptors is necessary for the induction of LTP of glutamatergic synaptic transmission (Calabresi et al., 1992, 2000; Pawlak and Kerr, 2008; Shen et al., 2008), whereas the induction of LTD requires activation of D2 receptors (Wang et al., 2006; Kreitzer and Malenka, 2007; Shen et al., 2008). Current experiments are aimed to determine how D1 and D2 receptors are differentially involved in LTP and LTD in the ipsilateral and contralateral pathways by interacting with GluN2B and GluN2A subunits.

Alterations in the ratio of GluN2B-containing NMDARs and GluN2A-containing NMDARs at synapses on MSNs correlate with dysfunctional motor behaviors, which has been thought to underlie striatum-related neurologic disorders such as Parkinson’s disease (PD) and Huntington’s disease (HD; Gardoni and Bellone, 2015). In an animal model of PD, partial lesions of dopaminergic fibers had no effect on GluN2B levels, but resulted in an increase of GluN2A levels, while full lesions reduced GluN2B levels without altering GluN2A (Picconi et al., 2004, Gardoni et al., 2006; Paillé et al., 2010). Normalizing the GluN2B/GluN2A ratio with a GluN2A-selective interference peptide, or by pharmacological activation of D1 receptors, restored synaptic plasticity in MSNs and improved motor function (Paillé et al., 2010). In an animal model of HD, a selective enhancement of GluN2B was observed in extrasynaptic NMDARs in striatal MSNs (Zeron et al., 2004; Milnerwood et al., 2010). Intriguingly, overexpression of GluN2B led to increased striatal neurodegeneration (Heng et al., 2009). Considering that altered NMDAR composition underlies striatum-related neurologic disorders, our observations of unbalanced GluN2B/GluN2A ratio at ipsilateral versus contralateral cortico-striatal synapses suggest that both pathways may have a distinguishing pathologic role in disease etiology and progression.

In summary, we demonstrate that the contralateral cortico-striatal pathway has higher levels of GluN2B-containing NMDARs than the ipsilateral pathway. Such distinct content of GluN2B and GluN2A subunits seems to contribute to the induction of different forms of LTP and LTD at ipsilateral and contralateral cortico-striatal synapses. These unexpected findings provide new insights into the mechanisms underlying NMDAR-mediated synaptic transmission at cortico-striatal synapses and have important implications for understanding striatum-related behaviors in healthy and diseased states.

## Synthesis

Reviewing Editor: Pablo Castillo, Albert Einstein College of Medicine Decisions are customarily a result of the Reviewing Editor and the peer reviewers coming together and discussing their recommendations until a consensus is reached. When revisions are invited, a fact-based synthesis statement explaining their decision and outlining what is needed to prepare a revision will be listed below. The following reviewer(s) agreed to reveal their identity: David Lovinger.

The reviewers agree your revised manuscript has significantly improved. However, they raised some concerns regarding your conclusions about dopaminergic signaling, which you should experimentally address. Alternatively, you may want to remove all experiments (and conclusions) on dopamine. Below, please find Reviewer 2's comments, which summarize both reviewers' take on your revised manuscript.

“The authors have improved most aspects of the paper with reasonable responses to the reviews. However, the lack of dopamine receptor agonist effects coupled with the antagonist effects is a major concern that was not addressed. It seems very unlikely that these results are due to a large dopaminergic tone in striatal slices. Effects of D1 and D2 agonists have been observed in many previous studies using this preparation (e.g. D2 agonist inhibition of dopamine release). Thus, there does not seem to be a tone that reliably occludes such effects. Desensitization during prolonged agonist application is a more likely explanation. However, the antagonist effects are the basis for the conclusions regarding the role of dopamine and quite simply these data are not convincing. To provide independent support for this point the authors need to use different antagonists (preferably with a different chemical structure), or show that the antagonist effects are lost in D1 and D2 knockout mice. While many receptor ligands are touted as ”highly selective” there are many instances of off-target effects in the literature, including for dopamine receptor-targeted compounds (e.g. Sah and Bean, Mol. Pharm., 1994). It might be simpler to remove the dopamine receptor experiments from the paper altogether. This would preserve the intriguing parts of the story (i.e. ipsilateral versus contralateral inputs, NMDA subunit roles, and effects on different MSN subytpes) without the uncertain conclusions about dopamine.”
